# Stable high volumetric production of glycosylated human recombinant IFNalpha2b in HEK293 cells

**DOI:** 10.1186/1472-6750-8-65

**Published:** 2008-08-27

**Authors:** Martin Loignon, Sylvie Perret, John Kelly, Denise Boulais, Brian Cass, Louis Bisson, Fatemeh Afkhamizarreh, Yves Durocher

**Affiliations:** 1 National Research Council Canada, Animal Cell Technology Group, Bioprocess Sector, Biotechnology Research Institute, 6100 Royalmount Ave., Montreal, QC, H4P 2R2, Canada; 2 Institute for Biological Sciences 100 Sussex Drive, Room 3100 Ottawa, ON K1A 0R6, Canada; 3 Department of Biomedical Engineering, Faculty of Medicine, McGill University, 3775 University Street, Montreal, QC H3A 2B4, Canada

## Abstract

**Background:**

Mammalian cells are becoming the prevailing expression system for the production of recombinant proteins because of their capacity for proper protein folding, assembly, and post-translational modifications. These systems currently allow high volumetric production of monoclonal recombinant antibodies in the range of grams per litre. However their use for large-scale expression of cytokines typically results in much lower volumetric productivity.

**Results:**

We have engineered a HEK293 cell clone for high level production of human recombinant glycosylated IFNα2b and developed a rapid and efficient method for its purification. This clone steadily produces more than 200 mg (up to 333 mg) of human recombinant IFNα2b per liter of serum-free culture, which can be purified by a single-step cation-exchange chromatography following media acidification and clarification. This rapid procedure yields 98% pure IFNα2b with a recovery greater than 70%. Purified IFNα2b migrates on SDS-PAGE as two species, a major 21 kDa band and a minor 19 kDa band. N-terminal sequences of both forms are identical and correspond to the expected mature protein. Purified IFNα2b elutes at neutral pH as a single peak with an apparent molecular weight of 44,000 Da as determined by size-exclusion chromatography. The presence of intramolecular and absence of intermolecular disulfide bridges is evidenced by the fact that non-reduced IFNα2b has a greater electrophoretic mobility than the reduced form. Treatment of purified IFNα2b with neuraminidase followed by O-glycosidase both increases electrophoretic mobility, indicating the presence of sialylated O-linked glycan. A detailed analysis of glycosylation by mass spectroscopy identifies disialylated and monosialylated forms as the major constituents of purified IFNα2b. Electron transfer dissociation (ETD) shows that the glycans are linked to the expected threonine at position 106. Other minor glycosylated forms and non-sialylated species are also detected, similar to IFNα2b produced naturally by lymphocytes. Further, the HEK293-produced IFNα2b is biologically active as shown with reporter gene and antiviral assays.

**Conclusion:**

These results show that the HEK293 cell line is an efficient and valuable host for the production of biologically active and glycosylated human IFNα2b.

## Background

Interferons (IFNs) are cytokines with major therapeutic applications based on their antiviral, antiproliferative, and immunomodulatory activities. Type I IFNs (IFNα/β) are massively produced in most cell types in response to viral and other microbial infections, and play a vital role in innate resistance to a wide variety of viruses [1]. The IFNα2 locus comprises three variants, IFNα2a, IFNα2b and IFNα2c, IFNα2b being the predominant one detected in human genomic DNA [2,3]. Some of the many diseases treated with IFNα2b, alone or in combination, include type B [4] and C hepatitis [5], several cancers such as melanoma [6-8], Kaposi's sarcoma [9], chronic myeloid lymphoma [10,11], and angioblastoma [12]. In the particular case of hepatitis C, a disease affecting over 170 million individuals worldwide, the combination of IFNα and the viral inhibitor ribavirin has become the standard treatment [13-15]. The rising incidence of certain cancers and viral hepatitis [16,17], in addition to ongoing investigations of novel therapeutic applications [18] are increasing the needs for human recombinant IFNα2b.

Human recombinant IFNα2b used in the clinic is synthesized in bacterial systems. When *E. coli *are grown in optimal conditions, a few grams (3 to 5) of recombinant human IFNα per litre of culture can be produced [19-21]. Bacterially produced recombinant human IFNα2b is misfolded and therefore requires refolding into its native conformation. Once purified and refolded, the recoveries are typically lower than 20% [19,20]. This refolding process also often results in loss of specific activity. In addition, bacterially produced recombinant human IFNα2b lacks the post-translational O-glycosylation present on the naturally synthesized protein. This non-glycosylated form of human recombinant IFNα2b has a shorter serum half-life than the glycosylated form [22]. The chemical conjugation of polyethylene glycol (PEG) molecules to the core peptide (pegylation) has improved the pharmacodynamics and pharmacokinetics of IFNα2b by increasing the serum half-life [23]. However, the pegylation of IFNα2b has been reported in some cases to reduce its biological activity [24]. It has also been shown that the size of PEG molecules and sites of attachment differentially interfere with the interaction and binding of IFNα2b to its receptor [25]. Nevertheless, the US Food and Drug Administration approved PEG-IFNα2b in 2001 and PEG-IFNα2a in 2002 for the treatment of chronic hepatitis C virus infection. Another common problem associated with the use of refolded and pegylated IFNα (PEG-IFNα) is the formation of neutralizing antibodies. Antibody formation against PEG-IFNα in HCV-infected patients has been associated with treatment failure [26,27]. In mice, the presence of contaminating partially unfolded IFN species appears to play a key role in the appearance of these antibodies [28]

Human and other mammalian cells are expression systems of choice for the production of secreted recombinant proteins such as antibodies, sometimes yielding up to hundreds of milligram to gram quantities of purified product per liter of culture [29-31]. However, the volumetric productivity of human cells for given proteins such as cytokines (i.e. IFNα2b) is often lower by several orders of magnitude. Originally, IFNα for therapeutic use was purified from the human lymphoblastoid Namalwa cell line following induction with Sendai virus. Despite the production of an IFNα with high biological activity, Namalwa cells were abandoned due to a limited productivity unable to satisfy an ever-growing demand. Other systems have been tested for the production of IFNα2b. For example, avian eggs have been used for the production of human recombinant IFNα2b [32,33], although the glycosylation pattern significantly differs from IFNα2b produced by human peripheral blood leucocytes. Glycosylated IFNα2b can also be produced in insect cells, but glycosylation is of the potentially immunogenic high-mannose type and lacks sialylation [34]. These limitations suggest that mammalian cells are preferable hosts for the production of fully glycosylated IFNα2b. Chinese hamster ovary (CHO) cells have been used for the production of various human recombinant interferons. Glycosylated and biologically active mouse IFNα [35] can be produced in CHO cells. Similarly, Rossman *et al *have reported the production of 120 μg/mL of IFNα2b using a glutamine synthase-amplified vector in the mouse myeloma cell line NS0 [36]. This is the highest level of glycosylated recombinant human IFNα2b produced in a mammalian system reported to date. *In vitro*, the biological activity of NS0-produced IFNα2b is very similar to that produced by Namalwa cells.

Here we have successfully engineered a non-amplified IFN-producing clone derived from the HEK293 mammalian cell line that produces hundreds of milligrams of IFNα2b per liter of serum-free media. The volumetric production of IFNα2b reproducibly exceeds 200 mg/L in batch culture and remains stable in the absence of selection for more than four months in culture. The purified IFNα2b is glycosylated and biologically active. Together these results demonstrate that cost-effective production and purification of glycosylated IFNα2b from human cells can be achieved.

## Results

### Generation of a stable IFNα2b-expressing HEK293 cell clone and production in fed-batch cultures

**Figure 1:**
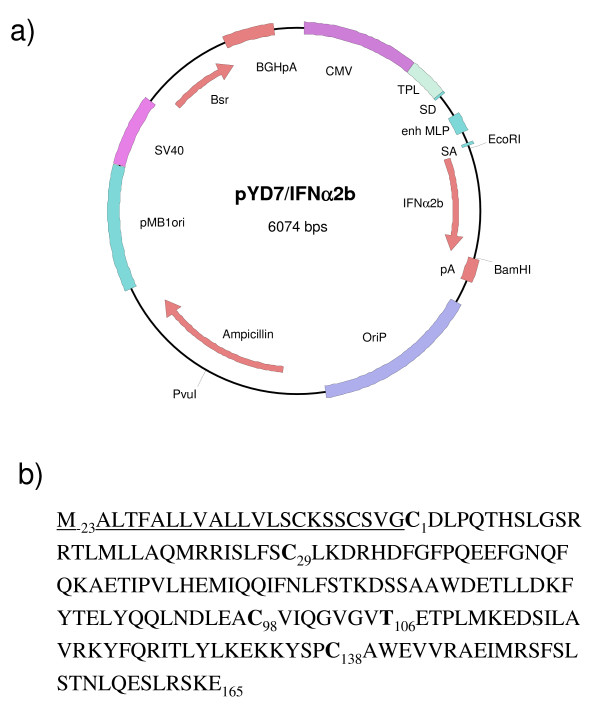
**Expression plasmid encoding human IFNα2b cDNA**. **A) **The pYD7-IFNα2b expression plasmid has been used to generate the D9 clone. (Amp) ampicillin, (Blast) blasticidin, (CMV) cytomegalovirus promoter, (enh MLP) adenovirus major late promoter, (IFNα2b) human codon-optimized sequence for human IFNα2b gene, (pA) polyadenylation sequence, (pMB1ori) bacterial origin of replication, (Puro) puromycin, (OriP) Epstein-Barr virus origin of replication, (SV40pA) simian virus 40 polyadenylation sequence, (TPL) adenovirus tripartite leader.**B) **Amino acid sequence of human IFNα2b. Signal peptide is underlined. The two intramolecular disulfide bridges are C_1_-C_98 _and C_29_-C_138_. The glycan-linked threonine (Thr_106_) is underscored.

**Figure 2:**
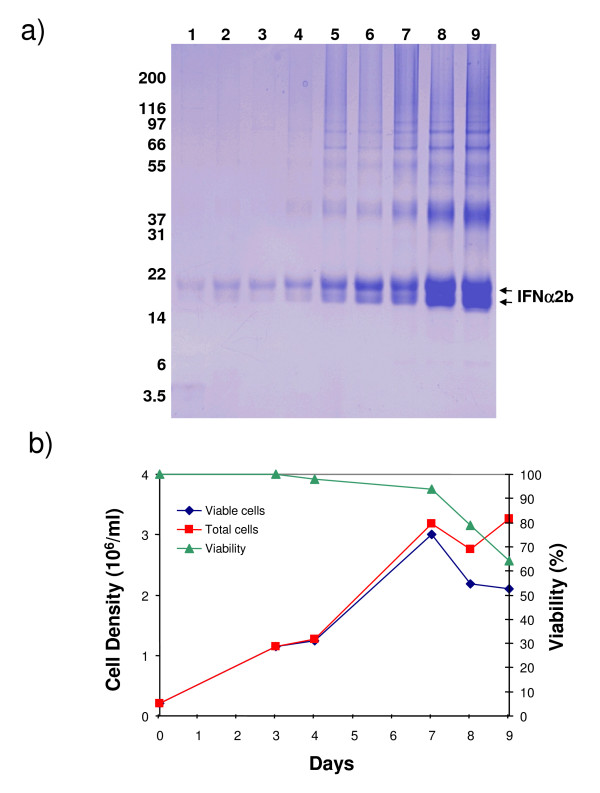
**Kinetics of cell growth and IFNα2b production from D9 clone in fed-batch culture**. D9 cells were seeded at a cell density of 0,25 × 10^6 ^cells per mL, fed with 0,1% TN1 the next day and sampled every day. **A) **Coomassie-stained SDS-PAGE analysis of the culture medium (20 μL) collected daily. **B)** Cell counts and viability were measured at the indicated times.

The expression plasmid pYD7 encoding the human IFNα2b gene codon-optimized for expression in human cells (Fig. 1A) is derived from the previously described pTT vector [37]. The signal peptide sequence, cysteine residues involved in intramolecular cystine formation, and the threonine of the consensus sequence for O-glycosylation of human IFNα2b are highlighted (Fig. 1B). The calculated molecular weight of the mature core protein (a.a. 24–188) of IFNα2b is 19,269 Da. In order to generate IFNα2b-producing cells, HEK293 were transfected with linearized pYD7-IFNα2b and selected in the presence of blasticidin. The D9 clone, which stably produces IFNα2b was isolated as described in material and methods. The production of IFNα2b with the D9 clone was performed in fed-batch culture. Daily samples from the culture media taken over a period of 9 days were analysed by Coomassie blue-stained gel (Fig. 2A). The gel shows that cell-derived contaminating proteins begin to accumulate significantly after day 5. A decline in cell viability was also notable after day 7 (Fig. 2B). Fed-batch cultivation was terminated and the culture medium harvested. It is noteworthy that early during production, HEK293-derived IFNα2b migrates with an apparent molecular weight of 2 kDa greater than its predicted mass calculated from the amino acid sequence (19,3 kDa), while at around day 4, a less abundant band of ~19,5 kDa appears. In order to ascertain that this band is also IFNα2b, N-terminal sequencing was performed on both products. The sequences obtained were identical and read NH_2_-C-D-L-P-Q-T, as expected for N-terminal sequence of human IFNα2b having a correctly processed signal peptide, therefore suggesting that heterogeneous posttranslational modifications may account for differences in electrophoretic mobilities of these two IFNα2b species.

### Purification of recombinant IFNα2b by cation exchange chromatography and analysis by gel filtration and SDS-PAGE

**Figure 3:**
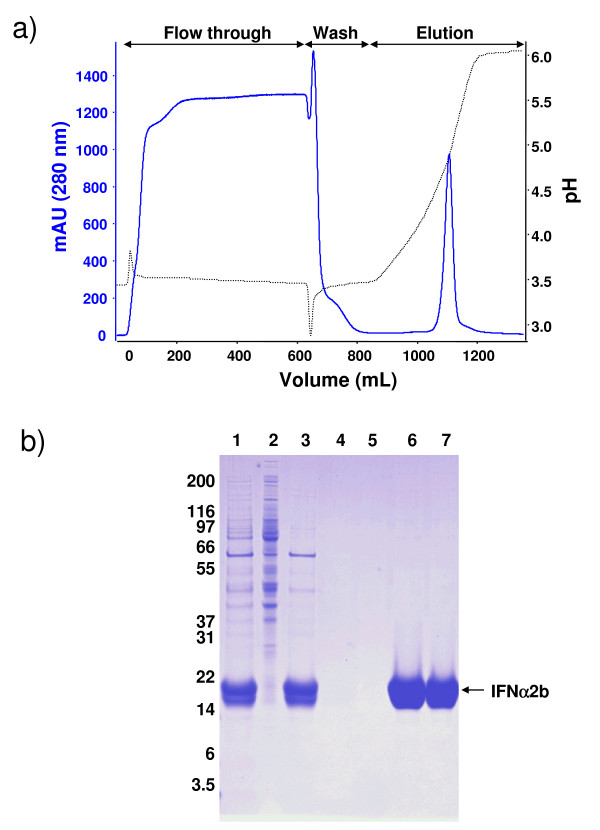
**Purification of IFNα2b by cation-exchange chromatography**. **A) **A typical chromatographic profile of a purification of HEK293-produced IFNα2b from a 400 mL fed-batch culture is illustrated. Solid line shows the 280 nM absorbance profile. Dotted line shows pH variations. IFNα2b elutes in a single peak between 1000 and 1200 mL. **B) **Coomassie-stained SDS-PAGE analysis of 20 μL samples collected at different steps of production and purification of IFNα2b. **1- **crude harvest. **2- **precipitate (equivalent to 200 μL of harvest volume). **3- **clarified harvest. **4- **flow through SO3^- ^column. **5- **wash SO3^- ^column. **6- **elution peak SO3^- ^column. **7- **desalted IFNα2b in PBS.

At the end of the production phase, the IFNα2b is purified as described in the Methods section. The IFNα2b eluted in a single peak at pH 4,5–4,6 from the cation exchange column (Fig. 3A). The electrophoretic profiles of proteins contained in the harvest, the acid precipitate, the clarified harvest and eluted fractions, are shown on a Coomassie blue-stained gel (Fig. 3B). The acidification step was selective in removing protein contaminants, as the concentration of IFNα2b in the clarified media was greater than 95% of that quantified in the harvest. The absence of IFNα2b in the flow through and in the wash suggests that IFNα2b strongly binds to the SO3^- ^column. According to a conservative estimate performed by densitometric analysis of the SDS-PAGE resolved purified material, the purity of IFNα2b exceeds 98% after the SO3^- ^column and the final desalting step.

**Figure 4:**
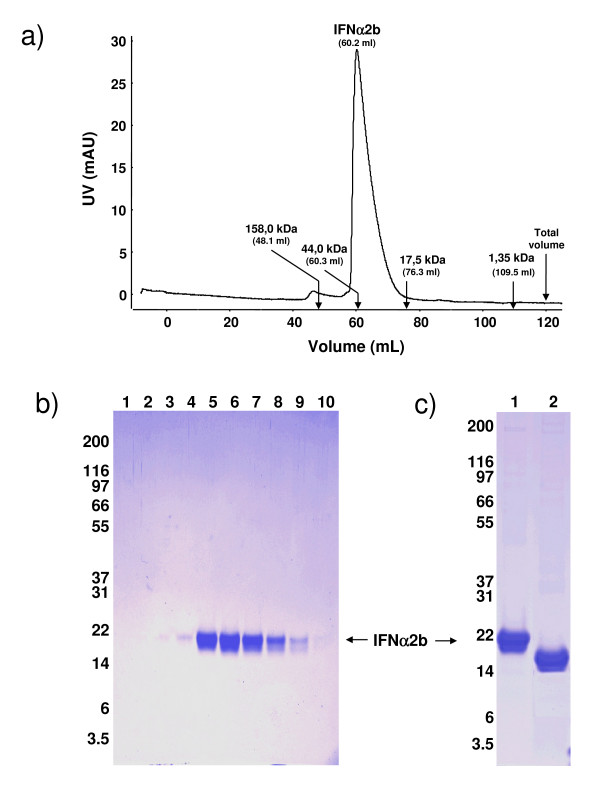
**Purified IFNα2b is not aggregated and forms dimers at neutral pH independent of intermolecular cystine formation**. Following a desalting step in neutral PBS, purified IFNα2b was analysed for dimer formation. **A) **Twenty mg of purified IFNα2b were analysed on a Superdex 75 HR16/60 column equilibrated with PBS at pH 7,0. The arrows and numbers above indicate the elution volumes of molecular weight standards eluted in the same conditions. Purified IFNα2b elutes in the same volume as ovalbumin, a 44 kDa globular protein. **B) **Coomassie-stained SDS-PAGE analysis of samples (20 μL) of each of the 10 fractions (4 mL) collected between elution volumes 40–80 mL. **C) **Coomassie-stained SDS-PAGE analysis of reduced and non-reduced IFNα2b from HEK293 cells.

Following desalting in PBS, purified IFNα2b was loaded on a Superdex 75 gel filtration column. The protein eluted as a single peak with elution volume identical to that of ovalbumin, a 44 kDa protein, indicating that purified HEK293-IFNα2b is not aggregated and suggesting that it may form dimers at neutral pH (Fig. 4A). A Coomassie blue-stained gel of IFN-containing fractions shows diffuse wide bands as observed with non-purified material (Fig. 4B). These species with different electrophoretic mobilities reflect glycosylation heterogeneity, which was later confirmed by mass spectroscopy and glycosylation analysis. Under reducing conditions, purified IFNα2b migrates as a major band of approximately 21 kDa and a less abundant band of lower molecular weight. Mass spectroscopy analysis indicates variations in the molecular weight between 19,922 and 20,659 Da (Fig. 6B). Under non-reducing conditions, IFNα2b migrates with an apparent molecular weight of ~17 kDa, a greater electrophoretic mobility typical of the presence of intramolecular disulfide bridges (Fig. 4C). The absence of dimers (i.e. ~42 kDa band) in non-reducing conditions indicates that the probable formation of dimers as suggested by gel filtration analysis is independent of intermolecular disulfide bridges. This non-covalent dimeric form of interferon has already been described and involves coordination of a zinc ion by two adjacent glutamic acid residues (E_41 _and E_42_) from each of two IFN molecules [38].

### The D9 clone produces hundreds of milligrams of IFNα2b per liter of culture that are efficiently recovered

IFNα2b in the crude harvests of fed-batch cultures was quantified by ELISA. The average concentration from two independent productions is 237 ± 11 mg/L, and this was increased to 301 ± 25 mg/L when glucose and glutamine feeds were added during production (Table 1). IFNα2b recovered from the SO3^- ^column measured by ELISA correlated well with measures obtained with a Bradford assay and by absorbance at 280 nm using IFNα2b molar extinction coefficient. The concentrations of IFNα2b measured by ELISA in the harvest and in the recovered fraction from the SO3^- ^column were used to determine the recovery. The mean concentration of IFNα2b shows that between 70 and 80% of the IFNα2b produced could be recovered, for two independent productions for each condition (Table 1). These results were comparable in terms of volumetric productivity and recovery to some productions of non-glycosylated IFNα2b performed in *E. coli *and in the methylotrophic yeast *Pichia pastoris *(Table 2).

**Table 1:** Quantification and recovery of HEK293-produced IFNα2b from two production schemes.

**Production scheme**	**IFNα2b mg/L (ELISA)**		**Percent recovery**
		
	**Culture medium**	**SO3**^-^**column**	
**1 feed**	237 ± 11	185 ± 3	79.5
**2 feeds**	301 ± 25	216 ± 11	71.8

**Table 2:** Overview of human recombinant IFNα2b production in prokaryotic and eukaryotic systems.

**Host**	**mg/L**	**Recovery**		**Purity %**	**Glycosylation**	Activity IU/mg*	**Ref.**
						
		**mg/L**	**%**				
**Prokaryotic**							

***E. coli***	5200	3000	58	ND	No	3 × 10^9^	[21]
***E. coli***	4000	300	7,5	ND	No	2,5 × 10^8^	[19]
***E. coli***	3500	600	12	100	No	ND	[20]
***S. lividans***	0,01	ND	ND	ND	No	0,4 × 10^4^	[55]

**Eukaryotic**							

***Pichia pastoris***	450	298	66,2	> 95	ND	1,9 × 10^9^	[56]
***Pichia pastoris***	200	ND	ND	ND	No	3.0 × 10^8^	[57]
**Tobacco BY2 cells**	0.02	ND	ND	ND	No	ND	[58]
**Insect Sf9 cells**	ND	ND	ND	ND	Partial (no sialylation)	2,3 × 10^8^	[34]
**Mouse NS0 cells**	120	ND	ND	ND	Yes	2 × 10^8^	[36]

* IFNα2b activity has been determined by inhibition of viral replication. Different viruses and hosts were used.

### IFNα2b produced in HEK293 is O-glycosylated, highly sialylated and biologically active

One of the major interests for producing IFNα2b in mammalian cells is to generate a glycosylated active protein. The apparent molecular weight of IFNα2b observed on SDS-PAGE suggests that IFNα2b produced in HEK293 undergoes post-translational modifications. There is also a less abundant product of around 19,5 kDa on SDS-PAGE.

**Figure 5:**
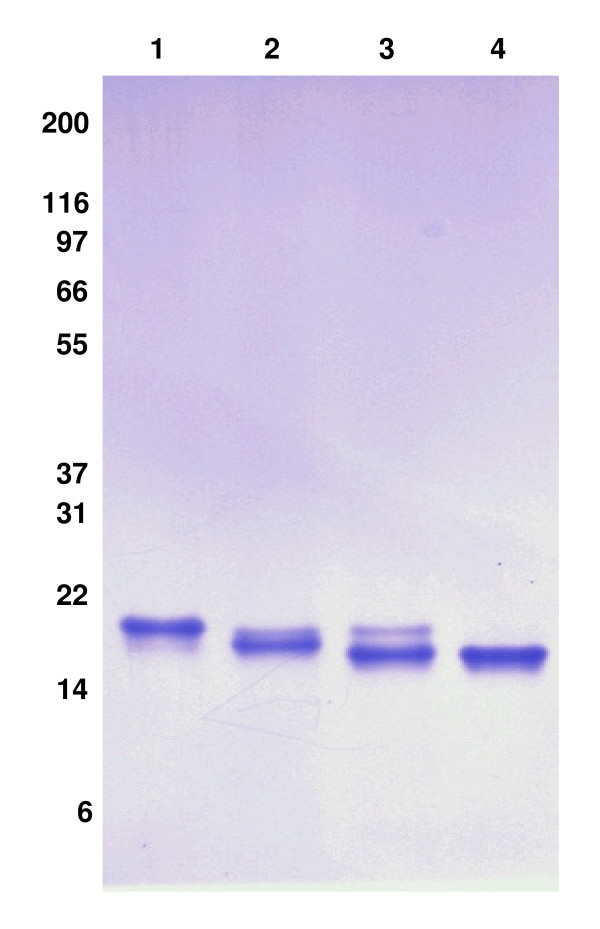
**HEK293-produced human IFNα2b is sialylated and O-glycosylated**. IFNα2b was deglycosylated as described in material and methods. **1- **10 μg of purified HEK-produced IFNα2b. **2- **10 μg of purified HEK-produced IFNα2b digested with neuraminidase. **3- **10 μg of purified HEK-produced IFNα2b digested with O-glycosidase. **4- **10 μg of purified *E. coli*-produced IFNα2b.

We next determined whether IFNα2b produced in HEK293 is O-glycosylated [39] and sialylated as previously reported for IFNα2b produced by human peripheral blood leucocytes [40]. We performed a sequential digestion of purified IFNα2b with neuraminidase and O-glycosidase to respectively remove sialic acid residues and O-linked saccharides. Each digestion successively increases the electrophoretic mobility of purified IFNα2b to generate a deglycosylated product that migrates as fast as non-glycosylated recombinant IFNα2b produced *E. coli *(Fig. 5). This suggests that IFNα2b produced in HEK293 cells is O-glycosylated and sialylated. Note here the quasi absence of the lower ~19,5 kDa product in the lane containing the non-digested IFN. We found that the majority of this product is lost during the purification process, as most of it remains bound to the column (data not shown). A minor band with lower electrophoretic mobility was still visible after glycosidases treatment, suggesting that this species might be Core 2 type glycan.

**Figure 6:**
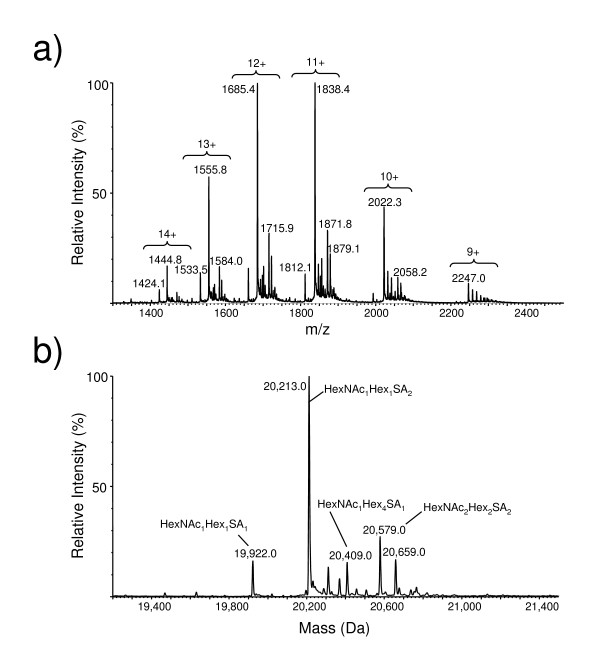
**ESI-MS analysis of the intact IFNα2b glycoprotein**. **A) **ESI mass spectrum exhibiting the glycoform profiles associated with each charge state of the protein and **B) **the glycoprotein molecule weight profile reconstructed from the mass spectrum in panel A. The most intense peak at 20,213 Da appears to be composed of the mature peptide chain plus a single core type-1 disialylated glycan (Hex_1_HexNAc_1_SA_2_).

**Figure 7:**
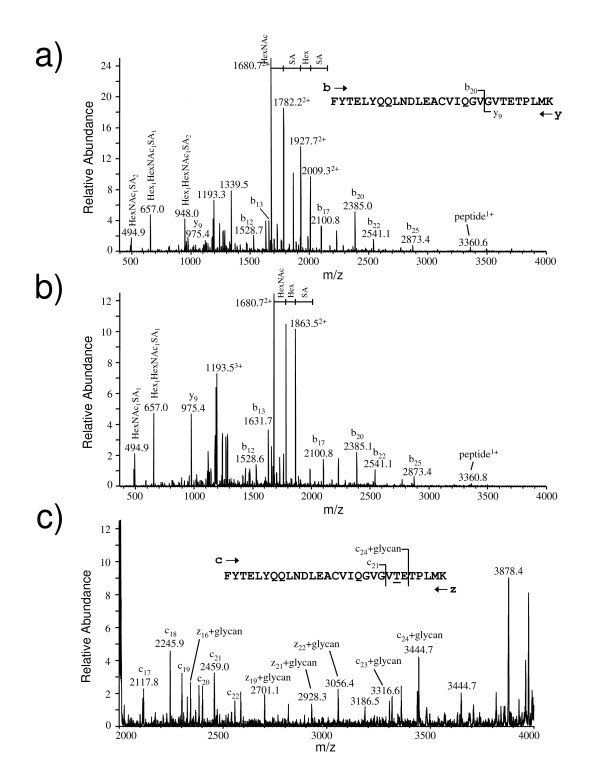
**CID and ETD analysis of the tryptic glycopeptides from IFNα2b**. **A) **CID-MS/MS spectrum of the triply protonated ion at m/z 1426.8 corresponding to the disialylated glycopeptide of T84-112. The spectrum is dominated by the sequential neutral loss of the glycan components from the doubly protonated glycopeptide ion. The principal b and y fragment ions arising from fragmentation of the peptide backbone are indicated in the spectrum as are the compositions the glycan oxonium ions observed m/z 494.9, 657.0 and 948.0, respectively. The sequence of the peptide is provided in the inset. **B) **CID-MS/MS spectrum of the triply protonated ion at m/z 1340.8 corresponding to the monosialylated glycopeptide of T84-112. Note that the neutral loss corresponding to a second sialic acid is missing from this spectrum as is the corresponding oxonium ion at m/z 948.0. **C) **ETD MS/MS spectrum of the triply protonated, monosialylated T84-112 glycopeptide at m/z 1340.8. The higher m/z region of the ETD spectrum contained the most informative fragment ions and is presented here. The c ion series indicated in the spectrum clearly identified the site of O-linkage as Threonine 106 of the mature protein.

A detailed mass analysis and glycosylation pattern of the purified IFNα2b was next performed by mass spectroscopy. Electrospray ionization (ESI) mass spectrum exhibiting the glycoform profiles associated with each charge state of purified IFNα2b is shown (Fig. 6A). The masses of the principal glycoform of this protein correspond to the mature IFNα2b peptide chain plus the glycans indicated (Fig. 6B). The most intense peak at 20 213 Da appears to be composed of the mature peptide chain plus a single core type-1 disialylated glycan (Hex_1_HexNAc_1_SA_2_). A MS/MS analysis of the tryptic glycopeptides confirms the composition of this glycan. The sialylated (mono and disialylated) glycoforms appear to constitute 75% of the total species. This percentage is likely to be underestimated, as some of the other peaks that cannot be assigned easily may be sialylated as well. The disialylated type 1 glycoform represents 50% of the total peak area while the monosialylated glycoform is 10% of the total. Using electron transfer dissociation, we also show that the glycan is linked to the expected threonine residue at position 106 (Fig. 7).

**Figure 8:**
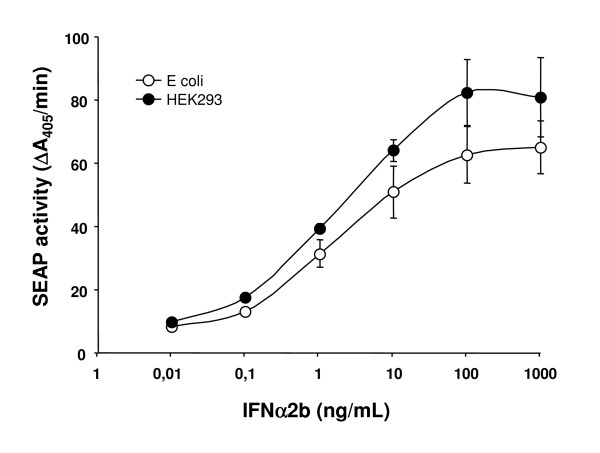
**HEK293-produced human IFNα2b is biologically active**. The biological activity of HEK293-produced human IFNα2b was assayed with a gene reporter assay and compared to *E. coli*-produced human recombinant IFNα2b as described in material and methods. The activity of the secreted alkaline phosphatase is plotted against the concentration of IFNα2b produced in the two hosts. Each point represents the average ± SEM of 3 experiments performed in triplicate.

Finally, we tested the purified glycosylated IFNα2b produced in HEK293 for *in vitro *biological activity in comparison to non-glycosylated form produced in *E. coli*. Using a reporter gene assay we show that HEK-produced IFNα2b is biologically active as it induces the production of a secreted alkaline phosphatase (SEAP) reporter enzyme under the control of the human ISG56 promoter (Fig. 8). This assay shows that HEK-produced IFNα2b is as active *in vitro *as bacterially produced IFNα2b. In addition, viral challenges using Vesicular stomatitis virus (VSV) on Madin-Darby Bovine Kidney (MDBK) cells or using Encephalomyocarditis virus (EMCV) on human A549 cells demonstrated very good antiviral activity of purified IFNα2b with titres ranging from 4.1 to 12.2 × 10^8 ^IU/mg for two independent batches (Table 3).

**Table 3:** Assessment of biological activity of HEK293-produced IFNα2b using two antiviral assays

**Antiviral assay**	**IFNα2b activity (U/mg)**			
	
	**HEK293 (1 feed)**	***E. coli***	**HEK293 (2 feeds)**	***E. coli***
**MDBK/VSV**	4.1 × 10^8^	3.1 × 10^8^	12.2 × 10^8^	4.0 × 10^8^
**A549/EMCV**	6.0 × 10^8^	3.9 × 10^8^	7.2 × 10^8^	8.1 × 10^8^

IFNα2b activity has been determined by inhibition of viral replication of vesicular stomatitis virus (VSV) in Madin Darby bovine kidney cells (MDBK) and of encephalomyocarditis virus (ECMV) in the lung carcinoma cell line A-549. Both antiviral assays were carried out by PBL InterferonSource. In each assay performed independently, *E. coli *IFNα2b was used as a positive control.

## Discussion

We describe here the generation of a HEK293 cell clone (D9) able to stably produce glycosylated human recombinant IFNα2b for culture periods up to 4 months in the absence of selection. The volumetric production per litre of serum-free culture can reach more than 300 mg/L, and is the highest volumetric production of IFNα2b reported for a mammalian system. We have further developed a rapid and reliable method for its efficient recovery and show that HEK-derived IFNα2b is O-glycosylated, sialylated and biologically active.

Gel filtration analysis of purified IFNα2b suggests that it may exist as a dimer in PBS at neutral pH. A zinc-dependent dimeric form of hIFNα2b as already been observed [38] and was also reported for the structurally homologous human IFNβ [41]. It is believed that interferon is biologically active as a monomer and the biological significance of zinc-mediated dimerization is currently unknown. The fact that our IFN is biologically active suggests that at the low concentration used in the bioactivity assays, it may exist in solution as a monomer.

To date, the production of recombinant IFNα2b and other cytokines in mammalian systems, particularly the development of clones stably expressing a cytokine of interest, has not been well exploited due to limitations in the volumetric productivity. One of the possible causes maybe that many cytokines exhibit strong anti-proliferative and cytotoxic activities on diverse cell lines [42,43], therefore strongly selecting against clones that show high cytokine expression levels. The D9 clone nonetheless grew almost as well as parental cells indicating that HEK293 cells can adapt to proliferate in the presence of high levels of IFNα2b. This adaptability of HEK293 cells to a growth inhibitory cytokine suggests that they may be suitable for the large-scale production of other interferons and cytokines.

To our knowledge, no comparable expression system exists in order to contrast the volumetric productivity of our HEK293 clone. However, this clone performs very well compared to other reported eukaryotic expression systems (see table 2 for an overview of IFNα2b expression systems). Nevertheless, a much greater volumetric production can be obtained from *E. coli *expression systems. Although we believe that the production capacity of HEK293 cells for IFNα2b can be improved, we doubt that such productivity can ever be achieved in mammalian cells, at least for a cytokine. It is obvious however that the difficulty in obtaining high recovery of refolded IFNα2b is still an important challenge with *E. coli*. In general, purifications of recombinant proteins from prokaryotes usually require extraction from inclusion bodies and complex refolding procedures, which reduce recovery yields [44]. Protein refolding is a critical step in the processing of biotherapeutics, as incompletely refolded species lower specific activity and may trigger an immune response. Antibodies to recombinant prokaryotic IFNα2b have been detected in HCV patients with acquired resistance to IFNα2b treatment [26,27], although it is not clear whether denatured IFNα2b played a role in this case.

Because the vast majority of biotherapeutics including growth factors, cytokines and antibodies are secreted proteins, mammalian systems, unlike prokaryotes, allow for production in perfusion as well as for the development of non-denaturing purification procedures. The first and foremost advantage of producing human recombinant proteins in mammalian systems is to generate proteins with the necessary posttranslational modifications required for full biological activity. N-glycosylation in particular, is often required for proper protein folding [45], protein-protein interactions, stability and optimal pharmacokinetics [46]. Although O-glycosylation is less critical for structure and function of proteins, it has been shown for example to increase the serum half-life of IGFBP6 by 2,3 folds over the non-glycosylated protein [47] and to protect against proteolysis [48]. In a recent randomized study, O-glycosylated IFNα2b was shown to have an increased serum half life in comparison to non-glycosylated IFNα2b [32]. We show here that human recombinant IFNα2b produced in HEK293 cells is O-glycosylated and extensively sialylated. Despite heterogeneity in the glycan structures, the nature and distribution of glycan moieties are quite similar to IFNα2b naturally produced by human leukocytes [40]. Approximately 50% of the purified protein is disialylated, while another 10% is monosialylated, in comparison to 50% and 30% respectively for leukocyte-derived IFN. As expected from its biochemical structure, we show that HEK293-produced IFNα2b has a biological activity comparable to that of non-glycosylated *E. coli*-produced IFNα2b by means of an *in vitro *reporter-gene assay, indicating that IFNα2b is not inactivated by the purification process. This was also confirmed by the high specific activity ranging from 4.1 to 12.2 × 10^8 ^IU/mg using two different batches of purified IFN and two antiviral assays.

## Conclusion

While additional studies are needed to determine whether HEK293-produced IFNα2b can offer advantages over non-glycosylated or pegylated IFN for *in vivo *applications, this work demonstrates that the HEK293 cell line is a suitable host for the high volumetric production of glycosylated human recombinant IFNα2b and potentially other cytokines.

## Methods

### Material

The expression plasmid was purified with a maxi-prep plasmid purification kit (Qiagen, Mississauga, ON, Canada). F17 serum-free culture media and blasticidin were obtained from Invitrogen (Carlsbad, CA). Pluronic F68 and glutamine were from Sigma-Aldrich (St. Louis, MO) and Tryptone N1 from Organotechnie (La Courneuve, France). Reagents for IFNα2b purification and electrophoresis include anhydrous citric acid and tri-Na citrate (EMD Chemicals Inc, Darmstadt, Germany), 0.45 μm filtering units (Millipore, Bedford, MA), NaCl (Sigma-Aldrich, St. Louis, MO), Fractogel* SO3^- ^(M) (Merck KGaA, Darmstadt, Germany), Econo-Pac^® ^10 columns (Bio-Rad Laboratories), Bradford Reagent (Biorad, Hercules, CA), 2 μm filters (Pall Corp, Ann Arbor, MI), NuPAGE Bis Tris 4–12% gradient gels, MES 20× buffer (Invitrogen, Carlsbad, CA), and Coomassie R250 stain (Sigma-Aldrich, St. Louis, MO). Trypsin (Promega, Madison WI), neuraminidase, dithiothreitol, iodoacetamide and guanidine HCl (Sigma-Aldrich, St. Louis, MO), O-glycosidase (Roche), Tris HCl, (Bio-Rad, Mississauga, ON), high purity acetonitrile, formic acid and ammonium bicarbonate (VWR International, Montreal, QC) and Centricon 3,000 MWL centrifugal filters (Millipore, Bedford, MA) were used for glycosylation analysis. The IFNα antibody and ELISA kit are from PBL InterferonSource (Piscataway, NJ, USA) and bacterially produced IFNα2b from Cell Sciences Inc (Norwood, MA, USA). pNifty2-56K-SEAP plasmid is from Invivogen (San Diego, USA).

### IFNα2b expression plasmid

The IFNα2b gene was synthesized with human-optimized codons (Geneart AG, Regensburg, Germany) according to the Genebank sequence no. AY255838. The synthetic cDNA was inserted as a BamHI/EcoRI fragment downstream of the cytomegalovirus (CMV) promoter into the pYD7 expression plasmid. This plasmid is a derivative of the previously described pTT vector [37] encoding the original functional elements in addition to a blasticidin resistance cassette.

### Engineering of a HEK293 clone stably expressing IFNα2b and fed-batch production

A HEK293 cell line constitutively expressing the EBNA1 protein of EBV (clone 6E) was used to generate IFN-producing clones. HEK293-6E and IFN-producing clone are grown in suspension in serum-free F17 culture media supplemented with 0.1% pluronic F68. Cultures were grown at 37°C and 5% CO_2 _under constant agitation (120 rpm). HEK293-6E were transfected as previously described [37] with PvuI-linearized pYD7/IFNα2b and selected in the presence of 2 μg/mL of blasticidin. The blasticidin resistant cells were next seeded into 96 well plates at 1 cell/well without blasticidin. After 3–4 weeks, emerging clones were expanded (in the absence of blasticidin) and tested for IFNα2b expression by dot blot. The selection of IFN-producing clones was based on the levels of IFNα2b expression and growth properties of the clones. The highest producers were amplified as suspension cultures and tested for IFNα2b accumulation over a 4 days culture. One clone, identified as D9, was selected because it is stably producing high IFNα2b levels while maintaining a high growth rate (doubling time of 26 hours^-1^). For IFNα2b production, cells were seeded at a density of 0,25 × 10^6 ^cells/mL in F17 antibiotic-free media in shaker flasks. Twenty-four hours post-seeding, the cultures were fed with 0,5% peptones [37,49] and left in the incubator for an additional 7–8 days. Optional addition of 20 mM glucose and 5 mM glutamine was performed 4 days post seeding where indicated.

### Purification of IFNα2b

The culture medium of a fed-batch culture was collected by centrifugation at 1000 g for 10 min. The supernatant was then acidified to pH 3.6–3.8 with 1 M citric acid. Acidification caused the formation of a precipitate which was removed by centrifugation. The clarified supernatant was then filtered on a 0.45 μm filtering unit. Purification of IFNα2b from the filtered supernatant was performed on an ÄKTA Explorer system (GE healthcare, Baie D'Urfé, QC, Canada). The supernatant was loaded at a flow rate of 10 mL/min on a Fractogel SO3-cation exchange column, previously equilibrated with 0,1 M Tri-Na citrate buffer pH 3,5 containing 0,35 M NaCl. Following a wash with 2 column volumes of the equilibration buffer, the IFNα2b was then eluted with a pH gradient. The pH of the mobile phase was increased from pH 3,5 to pH 6,0 with 0,1 M Tri-Na citrate buffer pH 6.0, plus 0,35 M NaCl. The fractions containing IFNα2b were pooled. An additional desalting step was performed on Econo-Pac^® ^10 columns according to the manufacturer's specifications. For the determination of glycosylation by enzymatic digestion, the purified IFNα2b was desalted in 0.1 M NH_4_HCO_3 _buffer pH 5 and lyophilized, whereas for bioassays, the purified IFNα2b was desalted in PBS and sterile filtered.

### Quantification and purity of IFNα2b

IFNα2b recovered from the SO3^- ^column was quantified by measuring absorption at 280 in a spectrophotometer, with a Nanodrop ND-1000 (Fisher Scientific, Montreal, QC, Canada), with a Bradford assay and by ELISA according to the manufacturer's protocol. The concentration in the harvest was measured with ELISA and used to calculate the percent recovery. To assess the purity level of IFNα2b, 3 μg were analyzed by SDS-PAGE followed by Coomassie staining.

### N-terminal sequencing and enzymatic determination of glycosylation of purified IFNα2b

As HEK293-produced IFNα2b migrates as two bands on SDS-PAGE, N-terminal amino acid sequences from both bands were obtained by automated sequencing performed at our sequencing facility. Enzymatic treatments with neuraminidase and O-glycosidase were performed to remove sialic acid and O-linked sugars respectively. Sequential digestions were performed in 50 mM phosphate buffer pH 5,0 on 100 μg of purified/desalted IFNα2b. Removal of sialic acid was done with 0.5 IU of neuraminidase for 1 h at 37°C followed by addition of 15 mU of O-glycosidase. Non-glycosylated recombinant IFNα2b from *E. coli *and glycosylated and deglycosylated HEK293-produced IFNα2b were resolved on SDS-PAGE in parallel to compare migration profiles. Migration profiles of glycosylated and deglycosylated HEK293-produced IFNα2b were compared to non-glycosylated IFNα2b produced in *E. coli*.

### Analysis of intact IFNα2b by mass spectrometry

The protein solution (~1 μg/μL in PBS buffer) was desalted by filtration on a 3 000 MWL Centricon filter and diluted to its original concentration with deionized water. The solution was adjusted to 20% acetonitrile, 0.2% formic acid just prior to infusion at 1 μL/min into the electrospray interface of a Q-TOF 2 hybrid quadrupole time-of-flight mass spectrometer (Waters, Milford, MA). The mass spectrometer was set to acquire one spectrum every 2 seconds over the mass range, m/z 800–2600. The protein molecular weight profile was generated from the mass spectrum using MaxEnt (Waters).

### Sequence analysis of the tryptic glycopeptides from purified IFNα2b

Purified IFNα2b was reduced, alkylated and digested with trypsin according to standard protocols. In summary, approximately 100 μg of the protein was dissolved in 1M Tris HCl, 6M guanidine HCl, pH 7.5 containing 2 mM dithiothreitol (DTT) and incubated at 50°C for 1 hour. The reduced cysteines were converted to carboxyamidomethyl derivatives using 10-fold excess of iodoacetamide over DTT. The protein solution was then concentrated on a 3 000 MWL Centricon and diluted to 100 μL using 50 mM ammonium bicarbonate. This process was repeated a second time. Trypsin (5 ug) was added to the sample, which was then incubated overnight at 37°C.

The tryptic digest was analyzed and fractionated by LC-MS using an Agilent 1100 HPLC system coupled with the Q-TOF2 mass spectrometer. Approximately 60 μg of the protein digest was injected onto a 4.6 mm × 250 cm Jupiter, 5 μm, 300 Å, C18 column (Phenomenex, Torrance, CA) and resolved using the following gradient conditions: 5% to 60% acetonitrile, 0.2% formic acid in 45 minutes, increasing to 95% after 50 minutes (1 mL/min flow rate). Approximately, 60 μL/min of the HPLC eluate was directed to the mass spectrometer while the remainder was collected in 1 minute fractions. The Q-TOF2 mass spectrometer was set to acquire 1 spectrum per second (m/z 150–2000) whilst cycling between a low and high offset voltage within the collision cell (10 V and 35V, respectively). This enabled the simultaneous detection of intact peptide and glycopeptide ions in the higher m/z regions (low offset mode) as well as the unique glycan oxonium ions in the lower regions of the spectrum (high offset mode). By screening the fractions in this manner it was possible to determine that only two of them (fractions 25–26 and 26–27 minutes, respectively) contained glycopeptides.

Glycopeptides were interrogated by collision induced dissociation (CID) to determine their amino acid sequence and glycan composition and by electron transfer dissociation (ETD) to identify the site of linkage. ETD preserves delicate modifications intact during the fragmentation process and is ideal for identifying the linkage sites of O-glycans [50-52]. The glycopeptide-containing fractions were infused at 1 μL/min into the electrospray ionization source of a LTQ XL linear ion trap (Thermo Fisher Scientific) capable of performing ETD. The CID collision voltage was adjusted for optimum production of peptide fragment ions from the multiply charge glycopeptide precursor ions (typically 25–30 V). ETD was performed using fluoranthene as the anionic reagent and with supplementary activation enabled. The optimal ETD reaction time for these glycopeptides was 350 msec.

### Biological activity

A SEAP reporter gene assay based on expression plasmid containing an IFN-inducible promoter (pNiFty2) was used to assess the biological activity of glycosylated HEK293-produced IFNα2b in comparison to non-glycosylated IFNα2b. HEK293 cells were transfected with the pNiFty2 reporter plasmid, which encodes the secreted embryonic alkaline phosphatase (SEAP) under the control of the human ISG56 promoter. Transfected cells were plated in 96 well plates at a cell density of 10^5 ^cells/mL and stimulated, 24 h post-transfection, with IFNα2b at the indicated concentrations. Following an additional 48 h period of incubation, the supernatants were collected and assayed for SEAP activity. The hydrolysis of paranitrophenyl phosphate (pNPP) was measured as a function of time to determine SEAP activity induced with IFN treatments, as previously described [53]. The SEAP activity is expressed as the increase in absorbance units at 410 nm per minute.

Antiviral assays were carried out by PBL InterferonSource (Piscataway, NJ) using Madin-Darby Bovine Kidney (MDBK) cells challenged by Vesicular stomatitis virus or human A549 cells challenged with encephalomyocarditis virus [54]. Cells were incubated with two-fold serial dilutions of IFNα2b standard (PBL InterferonSource), 293-IFNα2b, or control (Media). After 24 h incubation with the virus, cell viability was determined via crystal violet staining (570 nm absorbance). The sample titer (IC50), calculated by SigmaPlot software (SPSS Inc., Point Richmond, CA), was based on the 50% cytopathic effect of the assay. Units of anti-viral activity were based on the titer of a PBL lab standard, which was determined against the NIH reference standard for human IFNα2b (Gxa01-901-535).

## Abbreviations

EBV: Epstein Barr Virus; ESI: electrospray ionization; ETD: electron transfer dissociation; HEK: human embryonic kidney; IFNα2b: interferon alpha2b; PBS: phosphate buffered saline; SEAP: secreted alkaline phosphatase.

## Authors' contributions

ML performed production of IFNα2b, determined cell viability and growth curves in fed-batch productions, co-developed the purification method and purified IFNα2b, performed enzymatic deglycosylation, interpreted the data and wrote the manuscript. SP did ELISA assays and characterized the D9 IFN-producing clone. JK contributed the analysis of glycosylation by mass spectroscopy and editing of the manuscript. LB performed batch and fed-batch developments. DB performed the transfection and isolation of the D9 clone. BC co-developed the purification method and performed some batch experiments. FA tested IFNα2b for bioactivity. YD designed experiments, interpreted the data and revised the manuscript.
